# Clinique Medicale, ou Choix d'Observations recueillies à la Clinique de M. Lerminier, Médecin de l'Hôpital de la Charité, &c.

**Published:** 1825-10-01

**Authors:** 


*Clinique Médicale, ou Choix d'Observations recueillies à la Clinique de M. Lerminier, Médecin de l'Hôpital de la Charité, &c. et Publiées sous ses Yeux.* Par G. ANDRAL, Fils, Agrégé à la Faculté de Médecine de Paris, &c. &c. Deuxième Partie—Maladies de Poitrine. Paris, 1824. 8vo. pp. 591.

"Nulla est alia pro certo noscendi via, nisi quàm plurimas et morborum et dissectionum historias, tum aliorum, tum proprias, collectas habere, et inter se comparare."—MORGAGNI, *de sedibus et caus. morb.* Lib. iv. proem.

WHEN the discovery of the stethoscope was first vaguely promulgated, as the means of the physician to decide upon the healthy or morbid state of the thoracic viscera, by gravely listening, at the end of a piece of wood, there appeared to be something so very ludicrous, and, indeed, improbable in the idea that, in common with most of our brethren on this side the Channel, we were somewhat incredulous, and not a little sceptical respecting it.

A patient perusal of Laennec's work occasioned a speedy change in our first impressions. From it, we learnt that the first hint was derived from a well-known law in physics—of solid bodies communicating sound; and that the application of this property to the detecting of disease was owing to Laennec's being consulted in a case of diseased heart, in which, circumstances prevented him applying his ear to the patient's side, according to his usual practice.

Calling to mind the fact above alluded to, he rolled a quire of paper into a firm cylinder, and applying one end of it upon the præcordial region of the patient, and his own ear to the other, he heard the heart's action very distinctly. From the circumstance of space remaining in the centre of his paper cylinder, he accidentally learnt that the inflation of the air cells, by the act of inspiration, might also be heard through the thickness of the thoracic parietes.

Having established the facts—that the actions of the heart and lungs might be studied by auscultic instruments, he inferred that the examination of their phenomena would furnish much useful assistance in the diagnosis of their diseases; and accordingly he commenced his investigations, under favourable circumstances afforded by a Parisian hospital.

In the course of his investigations, he discovered the peculiar phenomena of the *voice* termed *pectoriloquism* and egophony: the former of which he considered to be almost pathognomonic of tuberculous excavations, and the latter, of pleuritic effusion. The natural varieties of *respiration* in infancy, manhood, and other cases:—the various shades of peculiarity, and the changes occasioned by disease, were investigated, as the French express it, with *rare sagacity:* and so many valuable diagnostic facts were collected, that there is scarcely a disease of the lungs or pleura, but what may be more or less elucidated by the exploration of the respiration. The details of the various *rattles* heard in the bronchia, have taught us much that is useful: and, lastly, from the facility with which he was enabled, by the stethoscope, to study the *rythme—impulsion—extent*—and different *sounds* in the *heart's action* he has furnished us with a set of signs by which we may determine upon some—detect many—and suspect almost all the various lesions the heart is heir to.

The original work appeared at Paris, in 1819, in two vols. 8vo. Of this, we gave an extended analysis in our number for January, 1820. Dr. Forbes's translation, published in 1822, was followed by a full notice, in our succeeding number.* Since that time, we have again recurred to the subject of thoracic diseases, percussion, and auscultation, in our review of Dr. Forbes's recent publication of excellent original cases, &c.† and we now return to some parts of the same subjects, for the fourth time, in the analysis of the work placed at the head of this article.

After the full exposition already given in the pages of this Journal, in the articles just referred to, it would be an useless

* Med. Chir. Rev. † Med. Chir. Rev. Jan. 1824, pp. 61—98.

repetition, if the present work contained nothing but what has already been explained. Fortunately, however, this is not the case. With respect to auscultation, we find much that is original, and much that establishes what was before doubtful, or corrects what was erroneous. Many obscure points of pathology are elucidated, and some valuable additions are made to our previous stores; and it is altogether such a rich fund of information upon thoracic diseases, that the place assigned to it in the library must be *next* to the work of Laennec, not as the *equal*, but as the *supplement*.

In the volume now under consideration, which commences with the diseases of the thoracic organs, the subjects treated of, are, (1) the different affections of the mucous coat of the bronchiæ —(2) inflammation of the pulmonary structure; and (3) of the pleura—all of them frequent in their occurrence, for the most part dangerous in their nature, and, more generally, within the reach of medical aid, than any other class of maladies of equally serious importance.

Our authors observe, that they are far from partaking in the opinion, that our knowledge has attained perfection upon the subjects embraced in this volume. On the contrary, they are firmly persuaded, that many of the most important points in the history of these diseases are still but imperfectly understood, notwithstanding the excellent works of the *French* writers, their cotemporaries, more especially those of Corvisart, Bayle, Broussais,* and Laennec. We extract their testimony respecting the utility of the stethoscope.

"Thanks to the wonderful discovery of auscultation by Laennec; the diagnosis of most of the thoracic affections may be frequently established with as much certainty as the most complicated luxation, or the simplest fracture. There are some cases of pneumonia, in which we may trace, by auscultation, the different 'phases' of the morbid alteration of the lung, and its return to the state of health, with as much precision as the eye can discern the different stages of cicatrization in a wound. There are affections of the heart, in which the nature of the organic lesion may be ascertained as accurately, by the ear, as the disposition of a cutaneous tumour by the eye, or the state of the cervix uteri by the touch. Nevertheless, clinical practice still presents many cases, in which the diagnosis yet remains obscure, and others, in which the nature of the affection is still at issue; it is to these that we shall more particularly direct our attention." *Preface*, vi—vii.

*Bronchitis*. The first chapter treats of bronchitis, an affection which, in one case, may be so slight as scarcely to deserve

* Histoire de Phlegmasies Chroniques, 1808.

the appellation of a disease, and, in another, so severe as to equal pneumonia, or phthisis, in importance. M. Andral states that, in a mild, recent inflammation, a small portion of the mucous membrane, in some of the primary divisions of the bronchia, is found of a red colour. When the inflammation has been more intense, the redness occurs, in a greater number of tubes, and also extends into the smallest ramifications. It frequently happens, that this red appearance is exactly limited to the bronchial ramifications of a single lobe of the lungs, most commonly, those of the superior lobe.

"When the inflammation has been *chronic*, the mucous membrane is found of a livid, violet, or brownish colour; and it is very remarkable, that, in some individuals, presenting all the symptoms of an inveterate chronic bronchitis, with purulent expectoration, we have found the mucous membrane of the air passages *scarcely reddened, or even perfectly white,* throughout its whole extent. Bayle has remarked the same fact, in the 49th observation of his work (on consumption). He relates, that the mucous coat of the trachea and bronchia appeared healthy, white, and not sensibly thickened, in a case of chronic bronchitis, simulating phthisis. 'This white state of the mucous coat,' he continues, 'is not unfrequent in chronic pulmonary catarrh.'" 3.

To controvert the opinion that, because there is no redness observable in these cases, there has been no inflammation, our author remarks, that absence of redness occurs, also, in other organs, where no doubt can be entertained of the existence of phlegmasia. In the serous membranes, coated by an exudation of lymph, it frequently happens that no change of colour, nor appreciable alteration of texture, can be discovered. The mucous coat of the intestines, even when sprinkled with numerous ulcerations, is often remarkably pallid, both as to the ulcerated parts, and as to the interstices between them; and, more than once he has found the mucous coat of the bladder, ureters, pelvis, and infundibula of the kidneys *quite white,* in individuals whose urine had long been purulent.

The following case affords an exquisite example of this state of the mucous membrane, and an excellent practical lesson upon the importance of distinguishing inveterate chronic bronchitis from consumption.

"A locksmith, aged 27, was admitted into La Charité, in December, 1821. For two years, he had been troubled with an habitual cough; he had never expectorated blood. At his admission, he was reduced to a state of marasmus; the expectoration consisted of separate, rounded, greenish patches of sputa floating in abundance of serosity: it was inodorous and sweetish to his taste. Respiration was rather short: he could lie flat down in bed, and *in every position.* Percussion gave a sound equally good all over the thorax: auscultation indicated mucous rattle in different places: there was *no pectoriloquism*. The pulse was not frequent in the forenoon, but became quickened towards evening—there were slight night sweats. Digestive organs undisturbed.

"What diagnosis could here be given? The stethoscope proved, indeed, (by the absence of pectoriloquism, *Rev.*) that no tuberculous excavations were formed in the lungs; but the other symptoms seemed to indicate the existence of numerous tubercles, in a state of incipient suppuration—(Decoction of lichen—'tisane de violette gommée-looch' —milk diet),

"The emaciation and general debility increased rapidly after his admission. Diarrhœa supervened—the intellectual faculties were disturbed—and he died in a semi-comatose state. Until the evening preceding his death, the sputa preserved the same appearance, and were undiminished in quantity.

"*Autopsy*. *Brain*—Sero-purulent infiltration of the cellular membrane placed beneath the tunica arachnoides of the convexity of the hemispheres: lateral ventricles distended by turbid serum. *Thorax*—Pulmonary tissue healthy, and moderately engorged. Internal surface of the larynx, trachea, and of the bronchia, (traced to their smallest ramifications) quite pale coloured throughout, and presenting no evident alteration. Right cavities of the heart distended with white fibrinous concretions. *Abdomen*—The digestive canal presented no other lesion than that of bright red-coloured patches, in the large intestines. Other viscera healthy." 4—6.

Cases like this we consider most valuable, as pointing out the danger of falling into a two-fold error; of mistaking the disease during life, and of being misled by the appearance of integrity in the respiratory organs after death. On the one hand, there was cough, attended with purulent expectoration—acceleration of pulse in the evening—night sweats and emaciation; indeed, the general symptoms of phthisis pulmonalis. On the other hand, dissection shewed the lungs free from tubercles, and the air-passages not apparently diseased.

The absence of pectoriloquism was sufficient to prove, during life, that no tuberculous excavations were formed in the lungs; and this opinion would, with us, have derived strong corroboration from the circumstance of the patient having no favourite position on lying down. For, since auscultation has enabled us to be more precise in our diagnosis of phthisis, we have almost invariably found that, after the suppuration of pulmonary tubercles, the patient can lie down comfortably, *only in one position;* and our investigations teach us directly the reverse of what is stated in Parr's Medical Dictionary, that the favourite position is upon the side in which the tubercles are suppurating, or upon the back, in a few cases, where both lungs are affected. The reason usually assigned by the patient for this choice of position is, that it quiets the cough, and that a change excites coughing and expectoration.

Whilst these circumstances would have led us to question the possibility of tubercles having suppurated, the mucous rattle heard in various parts of the chest, together with the negative evidence furnished by auscultation and percussion, of there being no other disease, would have directed us to the bronchia, as the seat of the affection, and source of the expectoration. Having established this point, we should not, with our author, have attributed the irritation of the air-passages to the presence of crude tubercles, because we have never known, and we scarcely believe it possible, for those parts to be kept in a state of irritation or inflammation by crude tubercles, for any considerable period, or even, for tubercles to exist long, where any part of the respiratory organs is inflamed, without their suppuration taking place.

We have, at the present time, a convalescent case of this kind under our care, to which we were called last February. A middle aged woman, who, for many years, has been troubled with cough and expectoration, was, at the time before-mentioned, reduced to a state of great emaciation and debility—the expectoration consisted of pale yellow purulent patches, floating in abundance of watery mucus—there were slight febrile exacerbations in the evenings, and night sweats—the position was on the right side, from habit; but she could lie upon the left. On percussion, the thorax sounded well every where, and more hollow than usual anteriorly. Respiration weak in places, and accompanied with sonorous and mucous rattles,—no pectoriloquism. Considering the air-passage as the seat of the disease, with possibly some degree of emphysema of the lungs, we treated the case, by the application of leeches to the trachea—blisters to the sternum—gave antimonials in minute doses, combined with a little digitalis, and attended carefully to the digestive organs. Under this treatment, she has improved much in all respects—has gained flesh and strength—and, we trust, will have her health finally re-established.

*Softening of the Mucous Membrane* of the bronchia is, according to M. Andral, much more rarely met with than in the intestinal canal.

---

*Ulceration* of the air-passages decreases in frequency as the part affected is situated lower down. In the larynx, chronic ulcerations are not uncommon; they are less frequent in the trachea, and of very rare occurrence in the bronchia. In the last named part, M. Andral has but twice seen ulcerations; once in the primitive division of the right lung, and once in the smallest ramifications. In the latter case, the ulcerations were very small, exactly circular, and of equal size—their margins were livid and elevated; the patient, who had also aneurism of the heart, had been tormented by frequent and very painful fits of coughing; the sputa were tinged with blood.

*Ulcerations in the Trachea* are generally small in size, and few in number: they rarely extend deeper than the mucous membrane; but a complete perforation of the trachea, from within outwards,* occasionally takes place. In one case, of this kind,

"There was a double perforation of the trachea and œsophagus, so that the two passages communicated freely with one another. The only symptoms of this tracheo-œsophagian fistula were a moderately great difficulty of deglutition, and a little coughing excited every time he swallowed. The patient pointed out the lower part of the neck as the seat of the difficulty of deglutition and cause of the cough." 10.

Perforation of the trachea from *without inwards* may be occasioned not only by aneurismal tumours, but also by tuberculous lymphatic glands. The latter lesion, M. Andral says, is not rare in children, although he has never observed it in the adult. In this way, tubercles of the bronchial glands are sometimes evacuated, but the patient rarely recovers, as tubercles almost always exist at the same time in the lungs.

*Thickening* of the mucous membrane of the bronchia is said to be one of the most frequent lesions produced by chronic bronchitis. It modifies the noise occasioned by the entrance of the air into the air-tubes of the lungs, and causes a peculiar rattle, which our author designates the *dry bronchial rattle*. It is the dry sonorous rattle, or "ronflement" of Laennec, to whose work we must refer our readers for further information. (*See Laennec de l'Ausc, t.* ii. 3. *Forbes's translation, p.* 319).

The thickening of the mucous membrane is sometimes so considerable as to *obstruct*, or even *obliterate*, the cavity of some of the bronchial tubes. M. Andral relates two cases of this kind, which the limits of this analysis do not permit us to

* In the original "*de dehors en dedans*," *evidently an error.*

detail. According to the degree of the obstruction, the stethoscope indicates diminution of the respiration, accompanied with dry rattles, or entire absence of respiration; whilst the sound, on percussion, always remains good. This combination of the signs, furnished by percussion and auscultation, will always distinguish the case in question from chronic inflammation of the lungs; but the particular lesion will not be ascertained, as the indications are nearly similar in emphysema, in obstruction from the pressure of external tumours, &c.

The subject next in order, is dilatation of the bronchial tubes, a lesion with which we were first made acquainted by the work of Laennec. M. Andral confirms almost all that writer has advanced respecting this very singular disease; and, as we now find, that the lesion is not one of the *rarest*, but only one that is *rarely observed*;—and, as it is also important to the auscultator, from the great resemblance its stethoscopic indications bear to those of tuberculous excavations:—we trust that a full account of this portion of the work will prove useful, at least to such of our readers as practise auscultation.

Laennec observes, "this affection of the bronchia is always produced by chronic catarrh, or by some other disease attended by long, violent, and often repeated fits of coughing. The hooping-cough, for this reason, is the most frequent cause of all. When the dilatation is confined to one or two of the bronchiæ, it produces no other inconvenience than an habitual cough, not very frequent nor severe, and a moderate degree of mucous expectoration; but, when a great number of bronchiæ are dilated, through their whole ramifications, the result is a chronic catarrh which lasts for life, and which, indeed, may sometimes shorten its duration, through means of the exhaustion produced by the dyspnœa, the severe and long-continued fits of coughing, and more especially, by the abundance of the expectorated sputa, which are of a greyish-yellow colour and altogether puriform. Sometimes, however, patients, affected with all these symptoms, and the degree of infirmity, the necessary consequence of these, live to an advanced age." (Forbes's translation, 78.) He further observes, (page 326) "We may regard it as certain, that, in the case of partial or general dilation of the bronchia, pectoriloquism will be found to correspond to the extent of the organic affection. *I am also confident, that in such case, the character of the voice, and the sound of respiration, will be such as to indicate that the phenomenon does not arise from an ulcerous cavity.*" We will now inquire how far M. Andral's cases confirm this conjecture.

*Case* 1.* A man, aged 62, who died of cancer of the stomach and chronic catarrh: a single tube in the middle lobe of the right lung was dilated to three times the size of the branch above it: auscultation indicated only the catarrhal affection.

*Case* 2. A house-porter, aged 66, with disease of the heart. *The voice resounded strongly*, resembling a *peculiar ringing* rather than pectoriloquism, in a space comprised between the clavicle and breast anteriorly, and in the supra-spinous fossa of the scapula posteriorly; and, in the same extent, he seemed to *puff* or *blow strongly* into the stethoscope at each inspiration. On dissection, the whole of the bronchiæ of the upper lobe of the lung were found dilated: wherever an incision was made, the open mouths of tubes almost as large as the principal branch were seen. The parietes of these dilated tubes were considerably thickened, and from space to space there were cartilaginous rings as distinct and as hard as at the division of the trachea.

*Case* 3. Chronic bronchitis, complicated with acute pleurisy of the *left* side. Over the infra-spinous fossa of the right scapula there was heard, at each inspiration, a kind of very strong *puffing* or *blowing* noise, (un sorte de souffle très intense) and on speaking, there was a *peculiar resonance* of the voice. The man recovered from the inflammation, and is still alive; the phenomena remain the same as when first discovered, and he is troubled with chronic catarrh.

*Case* 4. A peruke-maker, aged 46, with ulceration of the stomach. The respiration was weak, and the voice resounded strongly throughout the whole of the left side of the chest; there was pectoriloquism about the breast anteriorly, and just above the inferior angle of the scapula posteriorly. The expectoration in this case contained purulent patches, from which circumstance, together with the pectoriloquism, M. Lerminier considered the patient affected with phthisis pulmonalis. On dissection, the superior lobe of the left lung presented a cavity, the size of a walnut, the parietes of which were continuous with those of a small bronchial tube, and were evidently formed by the same tissue. In many points of this dilated portion, there were small openings leading into other bronchial tubes. In

---

* We here insert a very brief abstract of these cases, in order not to break the chain of analysis, referring the reader for a very full detail of them to the 2d No. of this series, (September, 1824,) page 451, *et seq.*—*Editors.*

tracing the bronchial ramifications throughout both the lobes of this lung, a considerable number of them were seen to enlarge suddenly to three or four times their former size, then contract again, and afterwards enlarge a second time.

*Case* 5. A few ramifications only were enlarged from space to space; their parietes being very thin and falling together, when cut into or slightly pressed. Although the patient had been frequently examined by the stethoscope and percussion, no suspicion of the lesion had been entertained during life.

From the above cases, and those related by Laennec, M. Andral has established three principal varieties of dilatation of the bronchia.

In the *first* variety, one or more tubes are more or less increased in capacity, throughout their whole extent; so that the tubes which arise from the 4th, 5th, or 6th division of the principal bronchus, are equal to, or of greater diameter than, the latter. M. Andral does not consider this form of dilatation to result from the simple passive distention of the bronchia, as the parietes are often found to be *thicker* than natural; the mucous membrane has more consistence and is denser; the fibrous structure is hard and resisting, whilst the cartilaginous matter is much more evident than usual. It is, he observes, a sort of hypertrophy affecting the structure of the bronchial tubes.

When *small* in degree or extent, this variety can scarcely be recognized during life. When *considerable*, it is announced by sufficiently characteristic symptoms. The *voice* resounds strongly under the instrument, as if there was a tuberculous excavation: there is frequently a mucous rattle or gurgle, analogous to that occasioned by partly evacuated tubercles: and, there is a puffing of the breath, or blowing into the cylinder, resembling that, which is frequently heard in tuberculous cavities.

In the *second* variety, the dilatation of the bronchia is not uniform; but one of the tubes is enlarged in some part of its course, more or less considerably, so as to produce a true accidental cavity, and compress the surrounding pulmonary structure. This variety is liable to be mistaken for a tuberculous cavity, but may readily be recognized by tracing the bronchial tubes.

The *diagnosis* will be more or less easy, according to the situation (whether superficial or deep-seated) and size of the dilated portion. When not deep-seated, there will be true pectoriloquism, and the characteristic puffing of the breath, during inspiration.

In the *third* variety, a tube is dilated at intervals, and presents in its course a series of dilatations and contractions. On incising the substance of the lung, it seems interspersed with a great number of white roundish swellings, remarkable for their colour, which depends upon the puriform fluid they contain. On opening these tumours with the point of a scalpel, we readily perceive, that they are cavities continuous with small bronchial ramifications, which (ramifications) are swollen from space to space, to form them. In this variety the parietes of the tubes appear thinner than natural, and M. Andral conceives, that the dilatations are the result of mechanical distention by the mucus, arising either from diminished elasticity, or from a true extenuation of the parietes. He says it is less rare in children than in adults; and he has frequently seen it at the Hospital des Enfans Malades. No *diagnosis* of this variety can be established, as it merely occasions rattle.

We have been thus full in our account of this lesion of the lungs, in order to guard the mere auscultator, lest he confound the two first varieties, with tuberculous excavations, an error to which he is always liable, from the similarity of the indications in the two diseases. Much experience in the use of the stethoscope and percussion, combined with accurate and minute investigation into the history and general symptoms, will, we believe, *frequently* enable the physician to distinguish the two cases; but the combination of these conditions is, we fear, at present too rare to authorise the expectation of such being a common occurrence. We, however, state with some confidence, our belief, that it is not impossible,—and whatever is possible ought always to be attempted.

Of the *treatment* of this affection, M. Andral says little; and but little that is useful, as far as we know, can be said. That the first and second varieties are incurable, there can be little doubt; the third, when the cause of the accumulation of mucus and of the cough is removed, may, perhaps, be within the reach of medicine, or, at least, of the vis medicatrix naturæ. In all the varieties, catarrh is a necessary accompaniment: to remove or alleviate this is, of course, a curative indication; and to effect this, we should treat it exactly as we treat chronic bronchitis, by keeping up a moderate action on the skin and a moderate action on the bowels. Mild purgatives, or rather aperients, and mild expectorants, with a little digitalis, if the pulse will bear it, an occasional blister to the sternum or a calefacient plaster, and even a few leeches to the trachea, will generally give relief; and above all, strict attention to the cloathing and diet is requisite. In one case, in which we believe the bronchia to be dilated throughout the greater part of the right lung, we have derived considerable benefit from the above means.

We now pass to the second section, which treats of the changes, secretions of the mucous membrane undergo from inflammation.

"The mucous membrane of the air passages," says M. A. "cannot be inflamed without its secretions being modified with respect to their quantity or their quality. The greater or less degree of obstruction of the bronchia, caused by the secretions, necessarily modifies the noise of natural respiration. Instead of the clear sound of 'pulmonary expansion,' (natural respiration) we hear a rattle, which evidently depends upon the displacement of a fluid by the column of air which enters the bronchia at each inspiration. This rattle, we designate by the generic term *moist* bronchial rattle, (the mucous rattle of Laennec.) It is far from being so characteristic as the *dry bronchial rattle* before described: sometimes it is confounded by insensible degrees with the crepitous rattle of pneumonia; and sometimes, especially when situated in the larger bronchia, it has a more or less perfect resemblance with the gurgle of tuberculous excavations. In the latter case, attention to the situation and extent of the rattle and other symptoms will elucidate the diagnosis with more certainty, than the character of the noise itself." 35.

"In the course of bronchitis, it sometimes happens, that the respiration and bronchial rattle suddenly cease to be audible in a certain extent of the lung; whilst the chest is as *sonorous* as usual on percussion: at the same time, the patient is seized with more or less dyspnœa. With Laennec, we attribute this sudden suspension of the respiration to the momentaneous obstruction of the bronchial tube, whose ramifications are distributed in the portion of the lung so affected. In such case, a strong fit of coughing, by expelling the tenacious mucus which causes the obstruction, restores the respiration as promptly as it had disappeared, and the dyspnœa ceases. In some very rare cases, the respiration is never re-established, the difficulty of breathing augments, suffocation becomes urgent, and death takes place from asphyxia. In this way a *slight bronchial inflammation* may be suddenly transformed into a *highly dangerous and rapidly fatal disease*." 40.

M. Andral relates two interesting cases which proved fatal from this obstruction. The first is that of a man who entered La Charité, with a rheumatic affection. The thorax gave a clear sound on percussion; respiration was very distinct throughout the left side; accompanied with mucous rattle in the superior and middle lobes of the right lung. No dyspnœa. Sometime afterwards he was suddenly seized, during a strong fit of coughing, with extreme difficulty of breathing: throughout the day and night following, there was orthopnœa and continual efforts to cough; next morning, imminent asphyxia, face swollen and of a violet colour, extremities livid, sensation of an immense weight pressing on the left breast and occasioning suffocation. The thorax sounded as well as before. Respiration was heard throughout the whole left side, and in the posterior part of the right; whilst, in the anterior part, from the clavicle to the breast, there was neither respiration nor rattle audible, notwithstanding that the chest was raised forcibly during inspiration. He died soon after. On laying open the bronchia, the point of the scissors encountered, at the origin of a large tube, a mass of concrete half-solid mucus, which closed up the passage like a plug. The tube thus obstructed, furnished the ramifications of the superior lobe of the right lung.

It is surprising, says M. A., that such serious symptoms should accrue, from an interruption to the passage of air in so small an extent of lung; whilst, in many persons whose lungs are almost wholly impermeable to air, life is sustained a long time, and frequently without much dyspnœa; but, in the latter case, the loss of permeability in the lungs is gradual; in the above instance, it was sudden. We may further remark, that if the bronchia had not been *carefully examined*, the case would have been regarded as *nervous asthma*; and the dyspnœa, occasioned by a mechanical cause, would have been considered a remarkable example of the metastasis of rheumatism to the lungs!

The second case is very similar; the obstruction was seated in the principal bronchial tube of the superior lobe; it consisted of a cylindrical mass of tenacious mucus, which extended into three or four ramifications.

M. Andral thinks, that when the attention has been once directed to the accident just described, the diagnosis will not be difficult to establish. "We may suspect," he observes, "obstruction of the bronchia whenever considerable dyspnœa supervenes suddenly in the course of simple bronchitis; the respiration, at the same time, being inaudible in a certain extent of the lungs, whilst percussion continues to give a clear or good sound in the part where the respiration is wanting. Emphysema is the only disease occasioning the same indications."

The curative indication is evidently to effect the expulsion of the obstructing matter. For this purpose, the efforts of vomiting should be tried. M. Andral also recommends the inhalation of the vapour of water, mercurial preparations, oxymel, squills, and remedies which are supposed to render the bronchial secretions more fluid, by increasing the exhalations from the lungs: and, as palliatives, he says, sanguineous depletions and revulsives are not to be neglected.

We shall close this chapter of the work, by extracting the remarks upon the sputa of bronchitis.

"To appreciate correctly the changes occasioned by inflammation, in the secretions of the mucous membrane of the bronchia, it is necessary to attend to the nature of this secretion, in different healthy individuals. In this respect, the bronchial mucous membrane presents as many varieties as that of the nasal fossa. Many persons, without having taken cold, expectorate during the day, and especially in the morning, a greater or less quantity of matter, which is sometimes thin and transparent, like the expectoration of the commencement of bronchitis, and sometimes thick, viscid, and opaque, like the sputa of chronic bronchitis.

"The expectoration commonly undergoes the following modifications in the different stages of acute bronchitis. At the commencement, the cough is *dry ;* and whilst it continues so, the disease is to be considered in its *first* stage. After a certain time, the length of which varies with the constitution of the individual and the remedies employed, every fit of coughing is followed by the expectoration of clear mucus, which is viscid and transparent, like white of egg, and when it is poured from one vessel into another, it runs together into an extremely tenacious mass. The tenacity and viscidity are increased in proportion as the irritation of the membrane is more considerable.

"When *fever* accompanies bronchial inflammation, the sputa have much greater viscidity during the febrile paroxysm; and, as the other symptoms of bronchitis are, at the same time, aggravated, an inexperienced practitioner might be deceived by this viscidity of the expectoration, and consider it as a sign of inflammation of the pulmonary structure. But if he examine the expectoration again, after the febrile paroxysm has subsided, he will find that the sputa have lost their viscidity.

"At other times, the expectoration is suppressed during the paroxysm; this indicates an increase in the affection of the mucous membrane.

"In some patients, the expectoration, towards the end of the sweating stage, which terminates the febrile paroxysm, consists of copious, thick and opaque sputa, similar to what occurs in the last stage of acute bronchitis. But this state is only transient; the patient soon expectorating again clear limpid mucus, the same as before the occurrence of the paroxysm. Thus, we see that, during a single paroxysm of fever, the expectoration may undergo all the modifications which it presents in the different stages of bronchitis.

"There is commonly more or less *froth,* upon the surface of the sputa; the quantity depends upon the facility with which expectoration is accomplished. If the patient has a violent fit of coughing, during which he breathes several times, before he can expectorate, the inspired air becomes intimately mixed with the mucus, filling the bronchia, and the expectoration then contains a large quantity of air, which forms a sort of lather on the surface.

"The sputa, in the first stage, are frequently streaked with blood, arising from small vessels which are ruptured by the efforts of coughing: the blood is *mixed* with the mucus—not *combined* with it, as in the rusty-coloured expectoration of pneumonia.

"The small *white portions of matter*, which are sometimes seen in the transparent mucus, do not originate in the lungs, but appear to be secreted in the numerous crypts of the pharynx and mouth. This matter has been erroneously looked upon as *the* "*debris*" *of tubercles*, and consequently, as one of the pathognomonic signs of phthisis.

"Whilst the sputa preserve the appearances above described, the symptoms of bronchial inflammation do not amend—the expectorated matter is still *crude*, according to the expression of the ancients: but, as the inflammation advances towards resolution, the sputa change their character, the mucus gradually loses its transparency, and becomes mixed with yellowish white, or greenish *opaque* masses, which are scanty at first, but continue to increase more and more, until, at length, they compose the whole of the expectoration. This change in the expectoration is ordinarily accompanied with a marked remission of the inflammatory symptoms; it indicates the resolution of the phlegmasia, that the *coction*, as the ancients termed it, is complete.

"The appearance and quality of the opaque sputa, expectorated towards the termination of acute bronchitis, are very variable.

"Notwithstanding the assertion made by writers, from Hippocrates to the present time, that the resolution of pulmonary catarrh cannot be considered complete, until the sputa become thickened and opaque, *we have seen* individuals attacked with intense bronchial inflammation, recover perfectly, although the sputa continued *crude* throughout the whole course of the disease; but these cases form *rare exceptions* to a general rule.

When acute bronchial inflammation passes into a chronic form, instead of terminating by resolution, the sputa retain the appearance they had in the last stage of the acute inflammation. They are opake, whitish yellow, or greenish; they sometimes adhere to the bottom of the vessel—sometimes float in transparent or turbid mucous fluid, or remain suspended in the middle of it. They are commonly inodorous, and appear insipid to the patient; and they are ordinarily expelled easily by slight coughing.

"It is frequently very difficult to distinguish the sputa of chronic bronchitis, from those appertaining to tuberculous degeneration of the lungs. We shall treat this subject in detail, when we come to the description of the expectoration of the latter disease.

"Bronchitis may continue for a long period, with expectoration similar to what is observed at the commencement of the affection. It is then an acute inflammation, indefinitely prolonged, and it is indicated, not only by the characters of the sputa, but, also, by the other symptoms, such as an habitual sensation of heat and constriction within the chest, painful and violent fits of coughing, increased temperature of the skin, &c." 51.

We now proceed to the second part of the work, which treats of pneumonia. The diagnosis of pneumonia is, for the most part, easy, and the treatment simple; nevertheless, there are some points in the history of the disease, to which the attention may be advantageously directed, some voids which require to be filled. Although it is ordinarily attended by symptoms which characterize it sufficiently, yet cases not unfrequently occur, in which all the usual signs are wanting, and the disease is *latent*.

Proceeding from the most simple and easy, to the most complicated and difficult cases, M. Andral has arranged his observations under five heads. In the first division, 29 cases, attended by all the usual characters of the disease, are detailed; these are subdivided into three sections, according to the *degree* of the affection, *viz*. 1, inflammatory engorgement. 2, red hepatization. 3, grey hepatization.

The second division contains 17 cases, in which one or more of the characteristic signs were wanting, It, also, is subdivided into three sections; the 1st, including cases in which the indications of auscultation and percussion were wanting; the 2d, those unattended by the signs furnished by the expectoration; and the 3d contains a single case, in which the signs of auscultation, percussion, and expectoration were all wanting.

The third division is devoted to the complications of pneumonia with other diseases. The fourth contains two cases of the rare termination of the disease in gangrene. Whilst the fifth is occupied with a "resumé" of the preceding divisions, or a general history of the disease.

Although M. Andral appears to have terminated his subject with what commonly forms the commencement, we think his arrangement highly judicious. By placing the general history in the last division, he leaves it to every one to form an unbiassed opinion from the perusal of the cases, which, we must say, are detailed with much care, candour, and ability; and, by afterwards collecting together, and arranging under their proper heads, the general facts which these cases afford, and placing them collectively before the reader, he enables him to scrutinize their accuracy, and to acquiesce or dissent from them according to his judgment.

The great number of cases which it would be necessary to detail, render it impossible to pursue the same plan in this analysis. We shall, therefore, select from the last division, M. Andral's description of the anatomical characters of the disease: —the indications of auscultation, to which M. Andral has made some valuable additions and some important changes; and some other miscellaneous observations. We shall then select a few cases to serve as illustrations of what has previously been advanced.

Laennec admits three degrees of inflammation of the parenchymatous structure of the lungs, *viz.* Simple engorgement, (engoument): red hepatization and grey hepatization. M. Andral employs the same terms because they are simple and in general use; at the same time he remarks, that the term hepatization is not accurate, as there is considerable difference between the consistence of *inflamed lung* and *healthy liver.*

"In the state which is commonly designated red or grey hepatization the pulmonary structure is remarkably *softened* and very *friable*; and it is only in some *much rarer cases* that it is found preternaturally *hard.* Is it right that these two different states of *softening* and of *induration*, presenting, as they do, differences in their symptoms, should be confounded under the same denomination? If we were not convinced of the extreme caution requisite in introducing new terms in medical language, we would propose the following nomenclature for the different stages of pneumonia. In *acute* inflammation we would admit three states, *viz. engorgement*, *red softening*, and *grey softening*; whilst in the *chronic* form, we would admit, in addition to the above, *red induration* and *grey induration.*" 306.

In the slightest degree of inflammation, that of simple engorgement, the parenchymatous structure is still crepitous, though in a less degree than natural; and it bears pressure and extension without tearing. On squeezing the lung, we perceive that the air-cells contain more liquid than air. The brown or mottled colour of the inflamed portion forms a strong contrast with the grey, or pale rose colour of the healthy part: if we incise the engorged parts, a large quantity of reddish foaming serosity runs out; and if, after being incised, the engorged parts are submitted to long continued pressure, so as to squeeze out all the fluid, they may be rendered as elastic as crepitous, and of the same colour as the healthy parts.

This state of simple inflammatory engorgement is only seen in cases of little severity; if the inflammation be at all intense, the structure of the lung is diminished in consistence, becomes friable, and breaks down readily, when pressed between the fingers. The fluid which runs out, when an incision is made, is less abundant and less frothy, and the structure has considerable analogy with the tissue of some spleens, which break down readily, without being reduced to pulp. This description applies to the state intermediate between the engorgement above described, and the red softening to be the next described; it is the passage from the first to the second degree of pleuro-peripneumony.

Whilst the lung is simply engorged, without other change of texture, it is sometimes difficult to decide, whether the engorgement is really inflammatory, or merely the mechanical result, either of the sanguineous engorgements with which the lungs are almost always charged, during the last moments of life, or of simple cadaveric engorgement. To distinguish these states, we must trust less to the colour than to the consistence of the lungs; and, if the structure be rendered friable, though in ever so slight a degree, it must be considered as inflammation. This increase of friability will be found, in almost all cases where inflammation has really existed, because it scarcely ever happens, that pneumonia occasions death, unless it be sufficiently intense in degree, to destroy, more or less, the ordinary consistence of the lungs.

From this simple engorgement—from this commencement of softening which we have just described—the inflamed lung advances gradually to another stage, which, at first sight, presents the appearance of liver, uniformly reddened by being gorged with blood. In this state, the red hepatization of authors —the lung is impermeable to air, and does not crepitate, nor swim in water; if we cut into it, a red fluid runs out, but it is not frothy, and is much less copious than in the preceding stage. Examined with a magnifying glass, the pulmonary tissue appears to be composed of a host of small red granulations pressed together; and when lacerated, the same granulations are frequently visible to the naked eye: it tears much more readily than in the natural state; the friability is so great, that it frequently requires only to be pressed slightly between the fingers, to break down the structure, and reduce it to a reddish pulp. Owing to the diseased lung not collapsing, as it does in the healthy state, its volume appears greater than natural.

M. Andral considers *red softening* to be a better term for this degree of inflammation, than *red hepatization*.

In a still further advanced stage, the pulmonary structure is dense, compact and impermeable to air, as in the preceding degree; and has a characteristic greyish, or ash colour. When examined with a glass, the same granulations are observed; only they are white or grey, instead of red. These granulations present many varieties with respect to arrangement, number, and size. In a given space, for example, there are sometimes but a small number, and they are either solitary or in clusters; sometimes they are grouped together in great numbers: whilst, at other times, there is only an uniformly smooth grey surface, without any appearance whatever of granulations. In this, as in the second stage, the parenchymatous structure is softened and very friable; the quantity of fluid it contains is sometimes so great, that, on making an incision, a greyish fluid, truly purulent, and always inodorous, rushes out. Sometimes the pus is not evacuated by a simple incision, but, on pressing the tissue gently, without breaking it down, the pus appears on the incised surface, in small drops, which seem to proceed either from the orifices of capillary bronchiæ, or from the granulations. In this degree, the more the lung is infiltrated with liquid, the more it is softened and friable: when pressed between the fingers it breaks down into a greyish pulp, from which circumstance it only requires the finger to be pressed upon a part, to form a small cavity filled with pus; and, the cavity thus formed may be mistaken for a recently formed abscess. M. Andral applies the term *grey softening* to this state.

In *chronic* inflammation, the lung presents the same changes above described, together with two other states, peculiar to the chronic form, in both of which, the pulmonary structure, instead of being softened and soaked with fluid, is hard and dry. In these states, the colour is sometimes red and sometimes grey, and M. Andral, therefore, designates them by the terms *red* and *grey induration.* He remarks that chronic pneumonia is a disease of rare occurrence, and consequently these states are but rarely seen.

It is common to find the three degrees of acute inflammation in the same lung.

*Grey softening* of the lung may be formed in a short space of time. M. Andral has seen a whole lobe in that state, on the fifth day of the disease.

With respect to the termination of inflammation in *abscess*, a lesion, which until late years was held to be of frequent occurrence, M. Andral agrees with Laennec, in ranking it among the *rarest* forms of the disease. He has met with one case only, in La Charité, that of a man who died on the 19th day, of peripneumony, in whom the abscess was situated in the middle part of the lower lobe of the lung; and, he has seen another specimen presented to the Royal Academy of Medicine, by Dr. Honoré, in which an abscess, the size of an apricot, was situated in the centre of an hepatized lobe. We have, ourselves, recently met with a case, in which four or five small abscesses were seated in the midst of hepatization.

Of *gangrene*, M. Andral relates two cases, and remarks that this termination of pneumonia is almost as rare as that of abscess. The ancients, he observes, have evidently described, under the name of gangrene, lesions of a different nature.

With respect to the seat of pneumonia, M. Andral gives the following numerical statement of cases.

| | Right Lung. | Left Lung. | Both Lungs. | Total. |
|---|---|---|---|---|
| In the Hospital of La Charité | 90 | 38 | 17 | 145 |
| In the Works of Morgagni, Stoll, De Haen, Pinel, and Broussais | 31 | 20 | 8 | 59 |
| Total | 121 | 58 | 25 | 204* |

With respect to the *part* of the lungs affected, M. Andral states that, in 88 cases, he found the *inferior* lobe inflamed in 47; the *superior* lobe in 30; and the *whole lung* diseased in 11. From this it is evident that Laennec's statement, respecting the extreme rarity of inflammation in the superior lobes,† is not to be depended upon, as we have ourselves ascertained in the course of our personal investigations. We notice this fact the more readily, because we think the discoverer of auscultation has attached too much importance to it, in regard to its proving the non-inflammatory origin of tubercles.

There is one *variety* in the seat of pneumonia which it is important to notice, because the diagnosis is frequently obscure. In this form the inflammation does not occupy a continuous portion of the lung, but is disseminated in numerous insulated points, which are surrounded by healthy tissue. These *partial pneumoniæ* occupy spaces, varying from the size of an orange to that of a nut or pea.

Inflammation of the air-tubes constantly accompanies inflammation of the parenchymatous structure; the mucous membrane is of a deep red colour, nearly equally so in the large and small ramifications.

In most cases there are traces of inflammation of the pleura, such as vascular injection, albuminous concretions, and serous,

* What explanation can be given of the greater liability of the *right* lung to be inflamed than the *left?* The same obtains even in greater proportion in phthisis: of 54 cases we have found *tuberculous excavations*;

In the right lung, and not in the left . . . . . . . . . . 22 cases.
In the left lung, and not in the right . . . . . . . . . 3
In both lungs . . . . . . . . . . . . . . . . . . . . . 31 *Rev.*

† "Rien n'est plus rare que de rencontre une inflammation bornée au lobe superieur de poumon." *Laennec de l'Auscultation, t.* i. *p.* 167.

purulent, or sanguineous effusion : it is on this account M. Andral generally employs the term *pleuro-pneumonia.* It does not follow, however, that every pneumonia is accompanied by pleurisy, "for more than once, after the most careful examination, the pleura has appeared perfectly healthy."

After some remarks upon the occasional causes of the disease, and a lucid account of its different modes of invasion, M. Andral proceeds to the consideration of the symptoms, the following of which he considers to be characteristic.

"Pain more or less marked in one of the sides of the chest—dyspnœa—viscid, sanguinolent sputa—dead sound on percusssion—modification of the noise of respiration—fever."

We shall extract a full account of the observations on auscultation.

"No sooner does the pain and difficulty of breathing come on, than the ear, applied to the thoracic parietes, recognizes a notable modification in the nature of the noise heard at each inspiration, and, as the inflammation advances, the noise undergoes fresh modifications, which indicate, with more or less precision, the situation and degree of the affection. The *voice* is also modified.

"At the commencement of the disease, whilst the lung is in the state of *simple inflammatorye ngorgement,* the *noise of respiration* in the affected part loses its clearness, and is more or less mixed with the *dry rattle,* which Laennec terms *crepitous,* from the resemblance it bears to the noise emitted by common salt, when thrown upon hot coals. It also bears considerable resemblance to the peculiar noise occasioned by folding or doubling a piece of parchment. The noise of *natural respiration* is always altered and obscured by this *rattle,* but is not always *entirely masked* or concealed by it. As the inflammation increases, the *rattle* becomes more and more pronounced, until, at length, it entirely conceals the *inspiratory murmur.* The presence of *crepitous rattle* indicates *engorgement,* or the first stage of inflammation ; and, so long as it continues, it shews that the inflammation (in great part at least) has not advanced beyond the first degree. From its greater or less intensity, and from its more or less strong admixture with the natural respiration, we may derive indications of the degree to which the engorgement extends, and whether it is passing into the state of hepatization, or otherwise. Whilst the noise of *natural respiration* predominates over the *crepitous rattle* we may conclude that the inflammation is slight ; but, if the *rattle* increases, and predominates in its turn, until, at length, it completely masks the respiration, we may be certain that the inflammation is advancing, and that it is passing on to the *second degree.*

"At a more advanced period the crepitous rattle gradually ceases to be heard ; and, if the *natural* respiration then returns, we know the disease is subsiding ; but if there be *no respiration* audible, or if the *natural* respiration is replaced by *another kind,* hereafter to be described, we may be certain that the disease is becoming more serious, and that the lung is *hepatized*.

"M. Laennec has well established the fact that, in many cases, when engorgement of the lungs is succeeded by hepatization, the ear applied to the chest *feels* the motions of the thoracic parietes, but does not *hear* any *respiratory noise*, either natural or pathologic. We have often verified this statement; but we have also frequently observed, in the same stage of the disease, *another very remarkable phenomenon*, which appears to have escaped Laennec's attention. In certain cases, where the lung is in the state of red or grey hepatization, the noise of respiration does not disappear, but is *modified* in a singular manner, and is evidently different from the natural kind. It seems as if a person, placed near the auscultator's ear, breathed forcibly through a brazen tube; there is, at the same time, a peculiar kind of *resonance* of the *voice*, wherever this kind of respiration is audible. The modification of the *voice* is not properly either *egophony** or *pectoriloquism*; it approaches more nearly to that form of resonance, which is observed in dilatation of the bronchia. Whenever cases, presenting this double modification of the voice and respiration, have proved fatal, dissection has constantly presented either red or grey hepatization, or pleuritic effusion: we have never observed it during life, but in cases where the very dead sound (on percussion) and the whole of the other symptoms indicated pneumonia in the second or third degree, or effusion of fluid into the pleura.

"The explanation of this modification of the voice and respiration seems easy. It appears to us to depend upon the air not being able to penetrate farther than the large bronchial tubes: and, for this reason, the phenomena are manifested, not only in pulmonary hepatization, but also where the lung is compressed by pleuritic effusion;—and, in short, whenever the air is prevented reaching the air-cells of the lungs. The cause of this peculiar respiration being well determined, we shall call it *bronchial respiration*, in order to distinguish it from the noise of natural respiration, which we designate by the terms, *noise of pulmonary expansion* and *vesicular respiration*. 335.

That M. Andral's description of the bronchial respiration and peculiar resonance of hepatization is accurate, we can vouch from our own experience. We have, in the course of our investigations, had many opportunities of comparing the relation which exists between these phenomena and the morbid appearances; and, from the important changes they are calculated to produce in the value of some of the other signs of auscultation, the information M. Andral affords us cannot be too widely disseminated.

---

* Egophony, or ægophony, is the proper orthography of this word, not hægophonism. Αἴξ has the smooth aspirate (')—it is, therefore, improper to prefix h; and for preferring the termination phony instead of phonism, there are several authorities in our language. e. g. symphony, euphony, cacophony.

Laennec, in treating of egophony, remarked, "that he was almost certain that it never existed in simple peripneumony;" —egophony is there used to signify resonance of the voice. His reasons for this opinion were founded upon the circumstance, that of 150 cases of inflammation which he had explored, he had never met with it; he adds "comme je les examinai tous très-attentivement, un phénomène aussi frappant que l'egophonie n'aurait guére pu echapper à mon attention, si réellement il eut existé chez eux." (*De l'Ausc. t.* i. *p.* 148.) He further observes that, "if peripneumony gave rise to egophony, the same effect would also be produced by induration of the lung from any other cause, and especially by the accumulation of tubercles, or the infiltration of tuberculous matter: and this, I can positively declare, is not the case; from the great number of consumptive cases I have examined, in whom the voice had been carefully explored, throughout the whole chest, a great many times, during the course of their mortal malady."

The first published facts, respecting this *resonance of the voice*, are to be found in Dr. Collin's work, (Des Diverses Méthodes d'Exploration de la Poitrine.) He relates, that in six cases of inflammation of the superior lobe of the lung, *pectoriloquism* existed. There is, however, only the positive evidence of one dissection; as four recovered and one died after some months illness, in the course of which phthisis supervened. M. Colin also considers resonance of the voice to be occasioned by the presence of numerous *crude* tubercles, when situated near to large bronchial tubes. M. Andral's numerous cases remove all doubt, and entirely supersede Laennec's observations.

Although our author justly observes that the *peculiar resonance* of the voice is not properly either *pectoriloquism* or *egophony*, still, its resemblance is so great, sometimes to the one and sometimes to the other, that it will not always be possible to discriminate. Nor will the *bronchial respiration* remove the difficulty; as *pectoriloquism* is accompanied with loud, *tracheal* breathing, whenever the tuberculous excavations communicate freely with the bronchia; and, in the points where *egophony* exists, "there is frequently tracheal or bronchial respiration," (*De l'Ausc.* 1, 354.) For these reasons, we would again strongly recommend the student not to rely solely upon the auscultic indications; but to study them in combination with the general symptoms, and the results of percussion. Where he has the opportunity of tracing the disease from its onset, auscultation will furnish him with precise and generally with sufficient information. He will trace the *crepitous rattle* progressively masking more and more the *natural respiration*, until, at length, the latter is entirely concealed; next follows the diminution and disappearance of the rattle itself; the *sound on percussion* diminishing and becoming *dead* or *fleshy* at the same time: then the *respiration* is either entirely *wanting*, or of the *bronchial* kind above described; if the latter, the *voice* also *resounds* strongly under the instrument. We say, that where the auscultator has traced these phenomena, in the course of an acute disease, he will find little difficulty; but when called in at an advanced period, and where there is complication with other affections, he will sometimes find even the resources of auscultation and percussion insufficient; and it may possibly require the minutest investigation of every circumstance to unravel all the intricacies and difficulties of the case.

"When pneumonia proceeds towards resolution, and begins to pass from the second back to the first stage of the disease, the *crepitous rattle* re-appears. At the same time, the *bronchial respiration* becomes less evident, and the resonance of the *voice* disappears gradually. The *crepitous rattle* diminishes in its turn, and is insensibly supplanted by the natural respiration or noise of pulmonary expansion.

"The *crepitous rattle* frequently remains in some points a long time after all the other symptoms of inflammation have disappeared: whilst this is the case, we may be sure that the pneumonia is not perfectly resolved; and we must be on our guard against a return of the acute form of the disease, or a continuance of a latent form, from whence disorganization may sooner or later result." 335.

"Whilst auscultation of the *diseased side* affords the different signs already enumerated; the *respiration* of the *healthy side* is heard with much greater intensity than in the physiological state;—as if it were necessary, for the *healthy* lung to receive a greater quantity of air in a given time, in order to supply the deficiency of the *diseased* one.

"This increased intensity of the respiration of *one side*, is of itself sufficient to induce suspicion of disease in the *other side*." 336.

"The quantity of liquid accumulated in the bronchia is sometimes so great, that the bronchial rattle occasioned thereby is loud enough to mask all the other auscultic phenomena.

"Lastly, there is a form of pneumonia in which auscultation teaches nothing with respect to the seat or degree. The ear perceives the noise of pulmonary expansion very clearly every where, but at the same time more strongly than natural. This happens when the inflammation occupies a circumscribed portion, situated at a distance from the surface of the lung, more especially a part of the *base* of the *centre*, or of the *root*." 337.

Our extracts already extend to so great a length that we can only make room for the following passage from the description of the expectoration.

"When pneumonia terminates in *gangrene* it is immediately indicated by the expectoration; at first the expectoration is a greenish liquid, then dirty grey, at times reddish, and *exhaling a fetid odour like that arising from the gangrene of external parts.*" 347.

We have experienced the correctness of this account to the great annoyance of our olfactory organ.

"With regard to the decubitus, nothing," says M. Andral, "can be more inaccurate than the opinion that the patient constantly lies on the *diseased side.* In the commencement of pneumonia there is scarcely one case in fifteen that affects this position; the others constantly rest on the back. It is in *certain pleuritic effusions,* and not in pneumonia, that the decubitus on the side is observed."

The observations upon the *treatment* are so truly *French*, and so little calculated to be useful with us, that we shall not waste our reader's patience nor our own labour in noticing them.

We shall now select a few cases.

CASE IX.—*Inflammatory Engorgement, passing into Red Hepatization.*

"A man, aged 45, entered La Charité January 21, 1820. On the 18th he had been all day exposed to cold and wet, in the evening he had a shivering fit, was burning hot throughout the night, and became delirious on the 19th, on which day he began to cough. On the 20th (3d day of the disease) no delirium, increase of the cough, slight dyspnœa, fever. When examined in the morning of the 21st he presented the following symptoms: Respiration accelerated, oppression, cough frequent, not painful, accompanied by expectoration of *rusty coloured, viscid,* and transparent sputa. *Crepitous rattle,* not entirely masking the inspiratory murmur, below *both clavicles* as far down as the breasts—in the *axillæ*—and in the *supra-spinous fossæ* of the scapulæ. *Respiration* elsewhere strong and clear. Thorax *as sonorous* as usual—decubitus on the back—pulse frequent and full—skin hot and dry, &c. &c. *Diagnosis*—Inflammatory engorgement of the summit of both lungs. (VS ad ℥xvj.— Sinapisms to the feet—ptisan—and glysters.

"Next day the *crepitous rattle* masked the respiration more completely, and extended to the right infra spinous fossa. The thorax, *on percussion,* was *less sonorous,* anteriorly; dyspnœa increased—and the *sputa* more viscid. The *inflammation* had therefore increased, and was advancing towards hepatization. (Two bleedings: one of 12 oz. in the morning, and one of 8 oz. in the evening; blood sized.)—Sixth day: same state, (blisters to the thighs.)—Seventh day: more and more oppression—*sound* below the clavicles *notably diminished. Weak crepitous rattle, without respiratory murmur,* in the same parts as before. Sputa red, united together into a gelatinous mass which adheres strongly to the vessel.—Eighth day:—delirium, death.

"*Autopsy.*—The *upper lobe* of each lung of a vermillion red colour, forming a strong contrast with the greyish hue of the other lobes. When incised, the superior lobes poured out a large quantity of red frothy serosity; their structure was more easily lacerated than the other lobes; and, although scarcely crepitous, they still floated in water. The rest of the parenchymatous structure was dry, excepting the posterior part, where there was slight cadaveric engorgement.

"Bronchial mucous membrane of the superior lobes reddened. Old cellular adhesions of the pleura—cavities of the heart filled with fluid black blood." 117.

This case requires but one comment, and that, as to the treatment. We believe there is scarcely a practitioner in the whole empire of Great Britain, who, with such accurate information, would have suffered his patient to die.

CASE XIX. *Red Hepatization.*

"A water-carrier, aged 58, was admitted into La Charité, March 9, having been troubled with acute pain under the left breast for three days. At his admission, there was *strong crepitous rattle* throughout nearly the whole inferior lobe of the left lung; in which extent, the thorax *resounded less on percussion* than on the right side. Although troubled with a constant desire to cough, he dare not cough out for fear of increasing the pain. *Sputa*, viscid and transparent, not streaked with blood—dyspnœa not great—pulse frequent and full—skin hot and dry—tongue whitish—thirst. (20 leeches to the side; V. S. ad. ℥xvj.—emollient ptisan.)

"On the 10th (4th day of the disease) dyspnœa increased. Sputa more *rusty* and very viscid. *Weak crepitous rattle*, not mixed with any *natural respiration*, in the whole inferior lobe of the left lung. The natural respiration in the left superior lobe, mixed at intervals with *slight crepitous rattle. Sound, on percussion, decidedly dead*, from the inferior angle of the left scapula to the bottom of the thorax.

"*Diagnosis.* From these signs, it was evident that the inflammation had advanced to the second degree in the lower lobe; and that the first degree was commencing in the upper lobe. M. Lerminier prescribed two bleedings: one of 20 oz. immediately, and the other of 12 oz. in the evening.

"Fifth day: same state.—6th day: extreme dyspnœa. Sputa very viscid, and bright red. *Bronchial Respiration* and peculiar *resonance of voice* on the level of the inferior angle of the left scapula, where the *sound* is very *fleshy* on percussion. On the same side, immediately above and below the spine of the scapula, in the axilla, and from the clavicle down to the nipple,—there is strong *crepitous rattle*, with slight admixture of *natural respiration*. The inflammation, therefore, was as bad as ever, and, notwithstanding that the venesection had been of no service, it was only by further depletions that its progress could be expected to be checked!—VS ad ℥xij!!

"7th day: The *bronchial respiration* had disappeared, which seemed to indicate an increase of hepatization. Other symptoms the same. (VS ad ℥viij.) Delirium in the night. Death on the 10th day.

"*Autopsy*. *Red hepatization* of the inferior lobe of the left lung: *sanguinolent engorgement* of the superior lobe: *albuminous concretions*, forming false membranes upon the left pulmonary and costal pleura, and about a glass-full of reddish serous *effusion*. Right cavities of the heart distended with clots of blood." 157.

In this case the indications of auscultation and percussion, enable us to trace the daily progress of the disease, with almost mathematical precision. He was first examined on the 3d day of the disease: there was *strong crepitous rattle* throughout the *inferior lobe* of the left lung, and the *sound* was *rather dull* on percussion. The disease, therefore, was limited to the inferior lobe, and still in the *first degree*, but fast verging to the *second*. On the 4th day, the inferior lobe was shewn to be hepatized, by the *dead sound* on percussion—the entire *absence of respiration*, and the *diminution* of the *crepitous rattle*: whilst the *slight crepitous rattle*, heard at intervals along with the natural respiration, shewed the commencement of *engorgement* in the superior lobe.

The *bronchial respiration* and *resonance of voice*, heard at the inferior angle of the scapula on the seventh day, was a certain proof of hepatization: and the extent in which the strong *crepitous rattle* with little admixture of natural respiration was audible, shewed the whole *superior lobe* to be very considerably *engorged*. The *bronchial respiration* disappeared on the seventh day; was not this occasioned by the effusion separating the lung from the thoracic parietes?

It is no wonder that the French surpass us in the knowledge of pathological anatomy:—the opportunities their *treatment* affords them being so much superior to ours. We are quite certain that, since we have used the stethoscope, we have never lost a case of simple pneumonia, however severe, to which we have been called by the 3d day. Our only opportunities of making post mortem examinations have been where the disease has been suffered to run its course, unchecked by appropriate remedies.

Case XXIX. *Red and Grey hepatization.*

"A sawyer, aged 67, troubled with a cough for five weeks, entered La Charité. For five days he had had *stitch* at the bottom of the left side, and had spit blood three days. At the first examination, there was *crepitous rattle* along with slight *respiratory noise* posteriorly on the left side. Percussion, on account of the pain it gave all over the side, could not be practised. Sputa *rusty* but little viscid. Pulse frequent and strong.

"The disease appeared not to have passed the first degree. (VS. ad ℥xvj.)

"6th day. *Crepitous rattle* posteriorly to the level of the inferior angle of the scapula: higher up there is *bronchial respiration* and a *peculiar resonance of voice*; seeming, as if the patient blew his words through the end of a tube: the *sound* is *dead* in the same part. Pain in the side almost gone.—Hepatization of the lung. (VS. ad ℥xvj. blisters to the thighs.)

"7th day. Sinking. *Bronchial respiration* audible in the same points as the preceding day: the *crepitous rattle* heard lower down has disappeared, and there is no noise of any kind. *Sound very dead* in the whole left side posteriorly.

"8th and 9th days. More and more great prostration—rapid sinking in the features of the face, without great difficulty of the breathing. Death in the night of the 9th day.

"*Autopsy.* The inferior lobe of the left lung, compact and easily lacerated, presented a mixture of *red* and *grey hepatization*, the latter being the most considerable; the rest of the lungs healthy and but little gorged with liquid. Nothing remarkable in the other viscera." 185.

This case requires no other observations than what we have made on the preceding ones. It affords a good example of *bronchial respiration* and *resonance of voice*.

CASE XXXVII. *Grey Softening of the Root of the Lung, not indicated by Auscultation or Percussion.*

"A man, aged 37, walked from Gueret to Paris, a distance of 92 (French) leagues, in six days, in very windy weather. On the 16th of March, 1820, the evening of his arrival, he was seized with a rigor, which lasted a part of the day and all the night following. On the 18th, he perceived an acute pain in the right breast. On the 19th and 20th, fever—cough without expectoration. He was admitted into La Charité on the 20th, and presented the following symptoms.

"Face calm, with a slight yellow tinge around the nose and orbits: sensitive and intellectual faculties undisturbed: considerable muscular power; position, on the back; breathing scarcely accelerated. Profound inspirations increase the pain, which still continues beneath the right breast. *Thorax perfectly sonorous* throughout, and the *respiration very distinct* on both sides. Cough frequent. Sputa yellow, viscid, united into a gelatinous mass, scanty; pulse frequent, small; tongue moist and clean; thirst; anorexia; abdomen supple and tardy; constipated (12 leeches to the side—emollient enemata and drinks).

"On the 22nd, the expectoration became aqueous and brownish, like the juice of plums, (blisters to the thighs).

"On the 23d, much worse. *Thorax* equally *sonorous* throughout. *Respiration* every where *audible* and *loud*. No expectoration. A few hours after the visit he vomited black matters, and sunk rapidly. He died at 5 o'clock.

"*Autopsy.* Both the lungs crepitous on their surface and engorged with frothy colourless serum. The structure, towards the root of the right lung, is reduced to a *greyish yellow pulp*, which breaks down easily under the finger, and oozes purulent sanies. Some points of *red hepatization* along the internal face of the summit of the lung." 207.

A very instructive case to auscultators, who ought always to be on the alert when the respiration is *louder* and *more distinct* than usual, in cases presenting the general symptoms of pneumonia.

Case LXIII. *Pneumonia terminating in Gangrene.*

"An organ-player, aged 28, drank, on the 25th of August, 1822, a large quantity of cold water, whilst he was in a state of perspiration. A few hours afterwards he was seized with a rigor; and, the same evening, he felt an acute pain under the right breast, which increased during the night. This continued throughout the 26th and 27th, on the latter of which days the sputa became bloody, and he was bled, with relief of the pain. On the 5th day of the disease he was bled a second time. Throughout the 6th, 7th, and 8th days, the sputa continued *rusty*, the cough frequent, oppression, and fever. The nine following days he remained in the same state, and used no other means than keeping in bed, and attending to his diet. He was admitted into La Charité on the 16th day from the invasion of the pneumonia, and was in the following state the next morning.

"Face pale and leaden-coloured: extreme weakness and emaciation: no trace of the pain in the side: he complained of dyspnœa—he lay flat upon the back, inclined a little to the right side: cough frequent: sputa reddish brown. The chest did *not resound on percussion* in the posterior and lateral parts of the right side; and there was *neither rattle nor respiration* in the same space. Pulse frequent: no heat of skin. M. Lerminier considered the case *pneumonia in the third degree.*

"He continued in the same state until the 21st day. In the morning, the sputa, which he had expectorated during the night, were rather fetid; and, in the course of the day, they changed to a dirty greenish grey colour, and became horridly fetid.

"Next morning the *dead sound* of the side had diminished, and there was a *marked gurgling* heard where the respiration was before inaudible. From this it appeared, either that purulent effusion, formed in the pleura, had opened into the bronchial tubes, or, what was more probable, that a communication was formed between one of the tubes and an ulcerated cavity resulting from gangrene.

"The following days the sputa became more fetid, and more characteristic of gangrene in colour. He sunk rapidly; he had profuse sweatings. Diarrhœa supervened on the 33rd day, and he died on the 42nd.

"*Autopsy.* The inferior lobe of the right lung excavated into a large cavity, with irregular brownish parietes, from whence exhaled a tainted gangrenous smell; it contained a dirty greenish grey semi-fluid, pultaceous mass; some large bronchial tubes opened into it, and it was only separated from the thoracic parietes by a very thin layer of pulmonary structure. Around the cavity, which would have contained an orange, the parenchyma of the inferior and middle lobes presented a mixture of red and grey hepatization." 228.

Cases of this kind are interesting, from their extreme rarity, and present but little difficulty. The only example we have seen was doubly interesting, from the circumstance, that the patient recovered, and is still alive. We are not aware that a similar instance is on record.

M. Andral's second case of gangrene occurred "in one of the most truly chronic examples of pneumonia" he ever witnessed. We cannot spare room to insert it.

Our analysis has already extended to so great a length, that we cannot give more than a table of contents of the last chapter which treats of pleurisy. In the first division, M. Andral treats of pleurisy occurring *without effusion*, and details three cases. These are valuable, because they form exceptions to the general rule of pleuritic inflammations being accompanied with effusion.

The *second* division contains the cases of pleurisy attended with effusion. The *third* is devoted to partial or circumscribed pleurisies, which are said to be very frequent and insidious. It is subdivided into three sections; the *first* of which includes three cases of *insulated* inflammation of the diaphragmatic pleura, and two cases of *diaphragmatic pleurisy* coexisting with inflammation of the pulmonary and costal pleura; the *second section* embraces *interlobular pleurisies;* and the *third,* inflammation of the *median pleura,* or mediastinum.

In the *fourth division,* three cases of *partial inflammation* of the pulmonary and costal pleura are detailed. The *fifth* embraces *double pleurisies,* with or without effusion. Whilst in the sixth division, M. Andral resumes the whole subject, and gives a general history of the disease, in the same way as he has done in the chapter on pneumonia.

In closing our remarks upon this volume, we must pay M. Andral the tribute of our warmest thanks for the candour, care, and able manner in which the cases are related, and for the valuable matter it contains. He deservedly ranks in the highest circle of his profession.

---

☞ The great length and importance of several Reviews in this Number, (occupying 206 pages of letter-press) compel us to defer a great mass of matter intended for the PERISCOPE till our next quarter. In our future Numbers we hope to make the proportion considerably more in favour of the latter department of the Journal.—*Ed.*

